# Economic downturn results in tick-borne disease upsurge

**DOI:** 10.1186/1756-3305-4-35

**Published:** 2011-03-15

**Authors:** Elinor R Godfrey, Sarah E Randolph

**Affiliations:** 1 Department of Zoology, University of Oxford, Oxford, UK

## Abstract

**Background:**

The emergence of zoonoses is due both to changes in human activities and to changes in their natural wildlife cycles. One of the most significant vector-borne zoonoses in Europe, tick-borne encephalitis (TBE), doubled in incidence in 1993, largely as a consequence of the socio-economic transition from communism to capitalism and associated environmental changes.

**Methods:**

To test the effect of the current economic recession, unemployment in 2009 and various socio-economic indices were compared to weather indices (derived from principal component analyses) as predictors for the change in TBE case numbers in 2009 relative to 2004-08, for 14 European countries.

**Results:**

Greatest increases in TBE incidence occurred in Latvia, Lithuania and Poland (91, 79 and 45%, respectively). The weather was rejected as an explanatory variable. Indicators of high background levels of poverty, e.g. percent of household expenditure on food, were significant predictors. The increase in unemployment in 2009 relative to 2008 together with 'in-work risk of poverty' is the only case in which a multivariate model has a second significant term.

**Conclusion:**

Background socio-economic conditions determine susceptibility to risk of TBE, while increased unemployment triggered a sudden increase in risk. Mechanisms behind this result may include reduced resistance to infection through stress; reduced uptake of costly vaccination; and more exposure of people to infected ticks in their forest habitat as they make greater use of wild forest foods, especially in those countries, Lithuania and Poland, with major marketing opportunities in such products. Recognition of these risk factors could allow more effective protection through education and a vaccination programme targeted at the economically most vulnerable.

## Background

Zoonotic infections, that circulate amongst wildlife and only infect humans accidentally, are not usually thought of as one of the ill effects of economic recessions. Tick-borne encephalitis (TBE), however, appears to be so in some parts of Europe. It is quantitatively and medically the most severe European vector-borne disease, caused by a flavivirus transmitted by *Ixodes ricinus *ticks in most countries in western, central and eastern Europe, and characterized by extreme spatial and temporal variability in its incidence. Dramatic upsurges in the early 1990s in most central and eastern European (CEE) countries have been related to environmental and socio-economic changes associated with the end of communist rule [1,2]. The former favoured increased abundance of infected ticks, while the latter promoted contact between those ticks and humans as they utilized tick-infested forests for either recreation or food harvest, depending on their economic status [3]. In western Europe, increases in incidence have been more gradual [4,5]; in Italy, for example, emergence has occurred in areas where forest types favour roe deer (*Capreolus capreolus*), the principal host for ticks [6,7]. Against this background, spikes in annual incidence occur sporadically in some countries for reasons that are not always apparent (e.g. in 2003 in the Baltic States - Estonia, Latvia and Lithuania - and Poland), but exceptional ones in 2006 in Switzerland, Germany, Slovenia, and the Czech Republic have been related to the unusual weather conditions during the second half of that year, apparently favouring both mushroom growth and outdoor recreation [8,9]. The response to such weather again varied with the national cultural and socio-economic contexts.

Low economic status has been identified as a significant risk factor for TBE infection in some parts of Europe. For eight CEE countries, the differential degree of TBE upsurge in the early 1990s was significantly positively correlated with contemporary poverty indicators, including the percentage of total household expenditure spent on food [1]. In Latvia, low income is linked to a greater probability of frequent visits to forests (tick habitat) for the purpose of collecting mushrooms or berries and a lower probability of being protected against TBE by vaccination [3]; furthermore, rural parishes with a low proportion of people with an economic activity have a higher probability of TBE cases [10]. Likewise, in Poland, replies to a questionnaire amongst 442 families involved in the harvest of non-wood forest products (NWFP) (principally mushrooms and fruit, but also fuel, decorative items and herbs) testify to the importance of income from the sale of these products for the economically disadvantaged [11]. The emergent prediction, tested here, is that the current economic recession might precipitate an upsurge in TBE incidence in certain countries where the response to poverty is more likely to include, amongst other things, activities leading to greater contact with tick-infested habitats and reduced protection through costly vaccination. Variations in both temperature and rainfall during the tick activity season have been shown to determine the weekly number of reported tick bites [3] and the monthly incidence of TBE [8], more through their impacts on human behaviour than on ticks. Therefore, we first tested the alternative hypothesis that any change in TBE incidence in 2009 was caused by unusual weather; it is discarded. Although we used time series data to derive our relative indices for 2009, we are actually taking a static view to make comparisons across countries.

## Methods and data

### Data sources

Information on the annual case numbers of TBE in each of 14 countries (with >10 cases per year) was acquired directly or indirectly from national Public Health Agencies via their web sites or the International Scientific Working Group on Tick-borne encephalitis (ISW-TBE). In addition to data at the national level, regional data for 2009 were available for most countries. For Hungary and Slovenia, data from the national diagnostic laboratories were used in favour of case numbers reported by doctors to Public Health Institutes, because the latter habitually suffer from under-reporting. These data are available from 1970 for most countries, but only those for the past six years are relevant to this present analysis. For Lithuania only, data on TBE incidence within different employment groups were also made available from the Lithuanian Public Health Agency. Figures for annual unemployment were extracted from Eurostat http://epp.eurostat.ec.europa.eu/cache/ITY_SDDS/EN/une_esms.htm; for Switzerland equivalent data were taken from the national statistics department http://www.bfs.admin.ch. These statistics were based on the International Labour Organisation's definition of unemployment: persons of a certain age who were not employed during the reference week, had actively sought work during the past four weeks and were ready to begin working immediately or within two weeks. They therefore represent responses to the immediate economic situation better than would figures for long-term unemployment. A variety of other socio-economic indicator variables for the period 2004-08 was extracted from Eurostat and the United Nations Development Program to explore any further socio-economic influences on TBE occurrence; these included GDP, expenditure on food (available for 2005), the Gini coefficient (a measure of inequality of income or wealth), poverty gap, at-risk of poverty, and in-work risk of poverty [12] (Table 1 for definitions). These socio-economic indices changed very little over this short period, so we used values for 2008 (apart from food expenditure) to indicate the most immediate background levels of poverty against which unemployment increased in 2009. Not all these additional data were available for Switzerland.

**Table 1:** Definitions of poverty indices

**GDP - gross domestic product**: The market value of all final goods and services produced within a country in a given period of time.
**Equivalised disposable income **(used below): Income adjusted for household size, achieved by assigning different members of a household the following weighting (taken from OECD): 1 for the first adult (>14 years), 0.5 for each subsequent adult and 0.3 for each person ≤14 years old.

**At-risk of poverty**: The share of persons with an equivalised disposable income below the at-risk-of- poverty threshold, which is set at 60% of the national median equivalised disposable income.

**Relative median at-risk-of-poverty gap **(abbreviated to **poverty gap**): The difference between the at-risk-of-poverty threshold and the median equivalised disposable income of persons below the at-risk-of-poverty threshold, expressed as a percentage of the at-risk-of-poverty threshold.

**In-work risk of poverty**: The share of persons who are employed whose household equivalised disposable income is below 60% of national median equivalised income.

To derive summary indices of the relevant weather during the tick activity season (typically April-October, giving TBE cases typically reported during May-November - [8]) monthly mean daily maximum temperature and daily precipitation (mm) were calculated from daily records downloaded from the European Climate Assessment web site http://eca.knmi.nl[13] for a representative site for each country over 1989-2009. Given the very similar weather patterns within any one country, and even between neighbouring countries, each site is taken as representative of the relative conditions in each year. For Hungary, it was necessary to use data from a meteorological station just across the national border in Croatia, but within the same TBE endemic region. To derive comparable measures of any unusual annual weather pattern, for each country the particular conditions for each month from April to October in each year were summarized as the deviations from the monthly means over the whole period (1989-2009), i.e. seven monthly anomalies per year per country.

### Analyses

**Figure 1:**
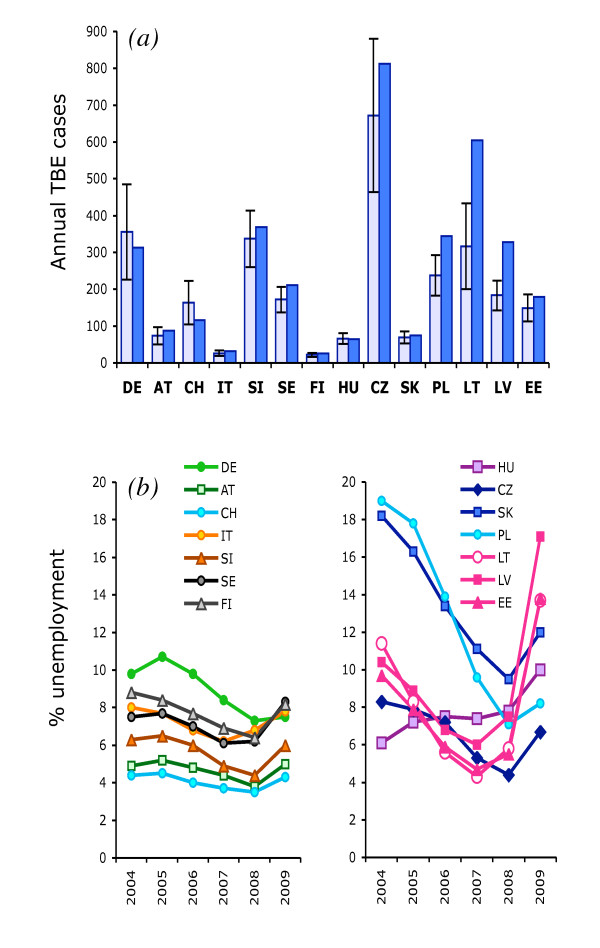
**Recent trends in tick-borne encephalitis cases and unemployment across Europe**. A) The number of cases of tick-borne encephalitis in 2009 (solid bars) relative to the mean numbers +/- 1StDev for 2004-2008 (light bars) for the same countries. B) Trends in the annual levels of unemployment (see text for precise metric) from 2004 to 2009 in 14 European countries. (Countries are divided into roughly geo-political groups simply for visual clarity.) DE Germany, AT Austria, CH Switzerland, IT Italy, SI Slovenia, SE Sweden, FI Finland, HU Hungary, CZ Czech Republic, SK Slovakia, PL Poland, LT Lithuania, LV Latvia, EE Estonia.

As the purpose of this study was to test for unusually high incidence of infection in 2009 compared with recent years, rather than to compare incidence between countries, raw case numbers were used without correcting for population sizes, which have changed very little over the period of interest. This avoids the unnecessary complication of the differential extent of TBE distribution within each country. As TBE incidence varies from year to year (see above and Table 2), case numbers in 2009 were compared with the mean level over the previous five years in each country (to give a '2009 TBE index'); this avoids any exaggeration in the measure of upsurge in 2009 due to randomly low numbers in 2008. This relatively short time series was chosen deliberately to maximize the uniformity of background forces operating across Europe, and specifically to avoid the long-lasting, slowly resolved effects of the major socio-economic transition that occurred in central and eastern Europe in the early 1990s [1], in order to test the specific hypothesis concerning the exceptional economic recession. Because of the highly variable patterns of recent trends in unemployment in each country (Figure 1b), change in unemployment levels was calculated as the ratio of 2009 to 2008 percentages to test the immediate effect of increased unemployment irrespective of recent trends. In fact, the ratio of unemployment 2009/2008 is highly correlated with the ratio for 2009/2004-08: correlation coefficient = 0.926, df 12, p < 0.001. The results of the analyses (below) were virtually identical (apart from very slight differences in values) whichever ratio was used.

**Table 2:** Tick-borne encephalitis cases recorded from 2004 to 2009 in those countries in Europe in which there were more than 10 cases per year

	**2004**	**2005**	**2006**	**2007**	**2008**	**2009**
Germany	274	432	546	238	289	313
Austria	54	100	84	45	86	88
Switzerland	132	208	245	111	123	116
Italy	31	19	30	17	34	32
Slovenia	264	375	446	266	334	369
Sweden	160	130	163	182	224	211
Finland	31	17	18	20	23	26
Hungary	89	52	56	62	70	64
Czech Republic	507	643	1029	546	633	812
Slovakia	70	50	91	57	79	75
Poland	262	174	316	238	201	345
Lithuania	425	243	462	234	220	605
Latvia	251	142	170	171	184	328
Estonia	182	164	171	140	90	179

To remove any co-variation in the weather variables taken from consecutive months, a principal components analysis (PCA) was performed on the monthly anomalies in temperature and precipitation, using the 'prcomp' function in the R statistical package [14]. Four PCA axes, that captured 76% of the variance in temperature and 71% in precipitation, were used for further analysis. Summary indices of the variation in weather by year and country were calculated as the Euclidean distance to the PCA centroid. As the conditions of normality were not met in several years, non-parametric t-tests (Mann-Whitney U-test) were used to compare the mean index across countries for each year against the mean for all other years to identify any unusual annual weather pattern. A comparable PCA for socio-economic conditions was not possible because there were too few indicators (8) relative to the number of countries.

Instead, univariate and multivariate normal linear regression analyses with normally distributed errors for the 13 countries with full socio-economic data were undertaken in the R statistical package [14]. All factors used in univariate analyses were then included in the multivariate analyses. Forward stepwise selection was used, i.e. to each explanatory variable each other explanatory variable was added until no further improvement in the model was achieved. Model selection was based on the Akaike's Information Criterion corrected for small sample size (AICc). PCA Euclidean distances for temperature and precipitation in 2009 were also included in these analyses.

## Results

**Figure 2:**
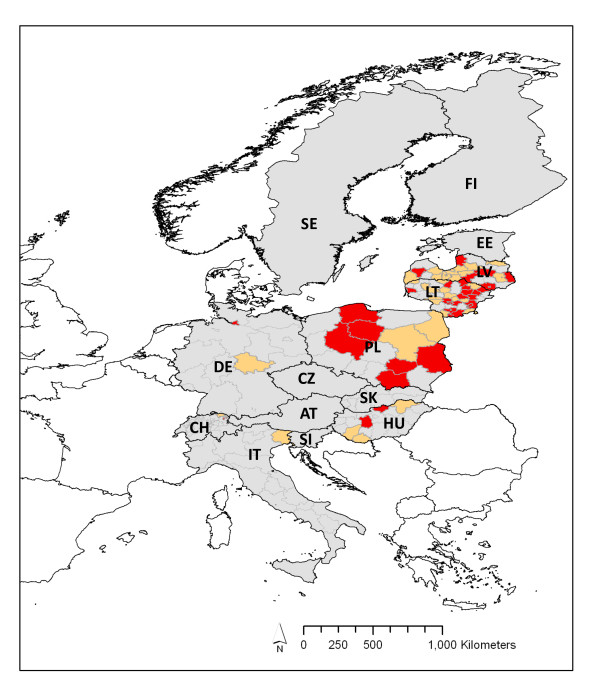
**Map of relative incidence of TBE in 2009 at the regional level in each European country with >10 cases per annum**. Sub-national regions shown in red, TBE in 2009 >1.5 × mean + 1 STDev for 2004-08 and exceeded the level in 2008; orange, TBE in 2009 1.01-1.5 × mean + 1 StDev for 2004-08 and exceeded the level in 2008; grey, TBE in 2009 no greater than mean + 1 StDev for 2004-08 or not exceeding the level in 2008. Incidence in 2009 was available at national, but not regional, level for Czech Republic, Austria, Slovenia, Estonia, Sweden and Finland. For country codes, see Figure 1 legend.

In Latvia, Lithuania and Poland, TBE cases in 2009 exceeded the mean plus one standard deviation (StDev) for the years 2004-08 (Figure 1a) and were also 1.7-2.8 times higher than in 2008 (Table 2). Although the increase in these countries was not statistically significant (i.e. not more than 2 StDev greater than the mean), it was considerable compared with the long-term normal year-to-year variation, and was much greater than for any other country. In Estonia, there were twice as many cases in 2009 as in 2008 due to the unusually low numbers in 2008, but the number for 2009 fell within the +/-1 StDev range for 2004-08. Case numbers in 2009 in all other countries fell within the +/-1 StDev range for 2004-08, and differed from 2008 by no more than 13% (apart from the Czech Republic, 28% higher). In Sweden case numbers in 2009 just exceeded (by 5) the mean + 1 StDev for 2004-08, but did not exceed those in 2008, due to the on-going upward trend since 2000. The geographical pattern is shown in Figure 2.

**Figure 3:**
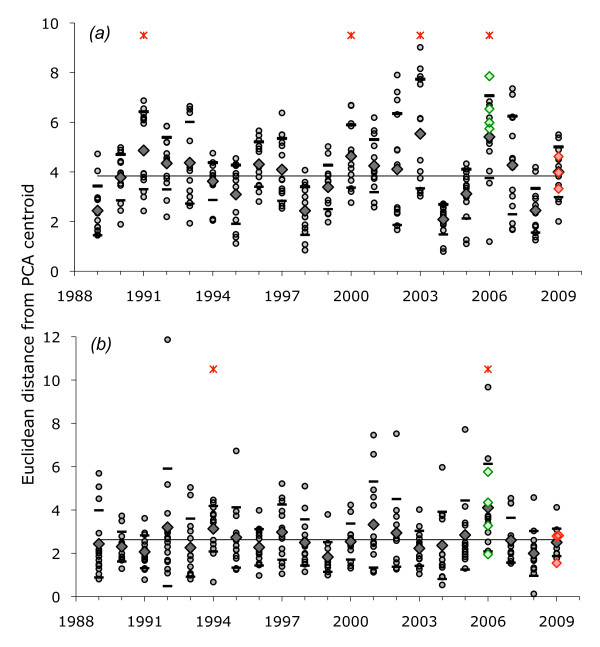
**Euclidean distances from PCA centroid of (A) monthly temperature and (B) monthly precipitation anomalies from the 1989-2009 means**. For each year, each of 14 countries (small grey dots) and the mean (black diamond) +/- 1 StDev (black dash) are shown. For 2009, Latvia, Lithuania and Poland are identified (red diamonds); for 2006, four countries that showed spikes in TBE incidence are identified (green diamonds). Red asterisks show years when Euclidean distances were significantly greater than the overall mean (black line).

The weather in 2009 showed no statistically significant difference from the average conditions for 1989-2009 (Figure 3). Furthermore, the Euclidean distances from the PCA centroid for Latvia, Lithuania and Poland (highlighted in Figure 3) were not consistently greater than for other countries. The weather in 2008, when the TBE virus-infected nymphal ticks that were active in 2009 would have been feeding as larvae and thus acquiring their infection, was even closer to the long-term average conditions. Only in 2006 were the patterns in both temperature and precipitation significantly different from the norm; for each specific country that suffered a spike in TBE incidence in 2006, the Euclidean distances in temperature were particularly large (>1 StDev above the overall mean for 1989-2009) (see discussion). In some other years, either temperature (1991, 2000, 2003) or precipitation (1994), but not both, showed significantly unusual patterns.

Unemployment increased in all countries in 2009, reversing the universal trend of steady decrease over the previous five years (except for Hungary, which showed an upward trend throughout this period) (Figure 1b). In all three Baltic countries, the increase had already started in 2008 (from the 3^rd ^quartile) and exceeded two-fold from 2008 to 2009. Noteworthy is the sharp decline in unemployment in Poland and Slovakia from very high levels in 2004, followed by relatively modest increases in 2009 (15% and 25%, respectively). Amongst the CEE countries, Slovenia and the Czech Republic showed overall patterns most similar to those in 'western' Europe (in the political rather than geographical sense), although both showed higher rates of increase from 2008 to 2009 (36% and 55%, respectively) than seen in the 'west' (3-34%).

Univariate analyses showed that national '2009 TBE indices' were significantly correlated with seven of the nine tested socio-economic indices, but not with the weather indices (Table 3). In all cases, the greater the poverty, the greater the 2009 TBE index. Because the dominant relationship with 'percentage food expenditure for 2005' was non-linear (2^nd ^order polynomial), the squared term of this variable was included in both univariate and multivariate analyses, and proved to be the most significant single correlate. This was not improved upon by adding any other variable to 'food expenditure^2^'. The list of significant variables indicates that background socio-economic conditions are as important as the sudden increase in unemployment in triggering activities associated with the increased risk of TBE. The high degree of co-variation between these socio-economic indices is revealed by the fact that the selection of 'in-work risk of poverty' as a second significant variable with 'unemployment ratio 2008/09' is the only case in which a multivariate model has a second significant term.

**Table 3:** Significant socio-economic variables to predict the degree of change in tick-borne encephalitis cases in 2009 relative to the mean for 2004-08

Explanatory variables	factor 1 p-value	factor 2 p-value	AICc
Food expenditure 2005^2^	**0.0013**		0.078
Food expenditure 2005^2 ^+ At-risk of poverty 2008	**0.0194**	0.1061	0.847
Food expenditure 2005^2 ^+ Gini coefficient 2008	**0.0322**	0.1239	1.180
Food expenditure 2005^2 ^+ In-work risk of poverty 2008	**0.0131**	0.1269	1.232
Food expenditure 2005^2 ^+ Poverty gap 2008	**0.0290**	0.2465	2.577
Food expenditure 2005^2 ^+ Ratio unemployment 2009/'08	**0.0317**	0.3138	3.026
Food expenditure 2005^2 ^+ Unemployment rate in 2009	**0.0249**	0.9508	4.407
Gini coefficient 2008	**0.0051**		3.102
At-risk of poverty 2008	**0.0075**		3.960
Poverty gap 2008	**0.0107**		4.743
Ratio unemployment 2009/'08	**0.0120**		4.985
Ratio unemployment '09/'08 + In-work risk of poverty '08	**0.0142**	**0.0162**	1.432
In-work risk of poverty 2008	**0.0138**		5.295
Unemployment rate in 2009	**0.0295**		6.931
GDP 2009	0.0898		9.222
Unemployment rate in 2008	0.8496		12.733
Temperature PCA Euclidean distance	0.8897		12.754
Precipitation PCA Euclidean distance	0.9471		12.773

Factor 1 and factor 2 refer to the predictor variables in the multi-variate analyses. Significant p-values are shown in bold.

Only some results of the multivariate analyses are presented to illustrate that second variables made significant contributions to the explanatory power of the models in only one case.

^2^For food expenditure, the squared term was used because a non-linear (2^nd ^order polynomial) relationship with TBE increase is dominant.

In all cases, n = 13 (Switzerland omitted for lack of some data); df = 3 for uni-variate, 4 for bivariate models.

**Figure 4:**
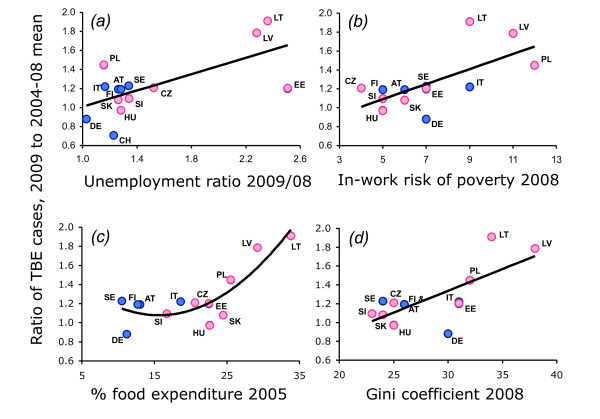
**Relationships between relative changes in TBE case numbers in 2009, unemployment and various ocio-economic indices**. A) Increase in unemployment in 2009 relative to 2008, *R*^2 ^= 0.437, p = 0.010. B) In-work risk of poverty 2008, *R*^2 ^= 0.437, p = 0.014. C) Percentage expenditure on food, *R*^2 ^= 0.745, p < 0.001. D) Gini coefficient 2008, *R*^2 ^= 0.524, p = 0.005. Countries as listed in Figure 1 legend: 'western' European countries (blue dots), central and eastern European countries (pink dots).

These conclusions are visually apparent from the univariate regressions (Figure 4) that highlight the outliers and exceptions to the overall effects of unemployment, most marked amongst CEE countries. In the three countries with exceptional increases in unemployment in 2009, TBE cases did indeed almost double in Lithuania and Latvia, but increased by only 20% in Estonia (Figure 4a). Conversely, the very modest increase in unemployment in Poland was associated with a 45% increase in TBE. This seems to be largely explained by the in-work risk of poverty (Figure 4b), which is highest in Poland (12) and much lower in Estonia (7 relative to a range of 5-9 amongst western countries). Tellingly, in Lithuania, the increase in TBE incidence was significantly greater amongst the unemployed (157%) than the employed (96%) (χ^2 ^= 4.57, p < 0.05), or pensioners (47%) (χ^2 ^= 6.66, p < 0.01).

With respect to socio-economics, as would be expected, 'western' European countries habitually fall in the lower left quartile of these plotted relationships and showed no significant effect of poorer socio-economic conditions on TBE incidence. Some CEE countries, notably Slovenia, the Czech Republic, Slovakia and Hungary also consistently fall there. Clearly, people's responses to the recession differ between countries, and only in some are these responses associated with activities likely to raise the risk of TBE infection, discussed below.

## Discussion

We report considerable increases in national mean TBE incidence in 2009 relative to average levels over the previous five years in three countries. Latvia, Lithuania and Poland, suffered 91, 79 and 45% increases in TBE case numbers, respectively, compared with <25% increase elsewhere and even 12-29% reduction in Germany and Switzerland. The propinquity of Latvia, Lithuania and Poland could suggest spatial autocorrelation that would undermine the statistical analyses, but this is highly unlikely in this case. Contiguity *per se *does not result in similar patterns of TBE incidence or change in incidence [1-3,8]; TBE is not contaminative and the vector ticks do not fly. Estonia is also contiguous with and part of the same geographic and politico-historical group as Latvia and Lithuania, but shows neither the same current poverty levels nor TBE spike in 2009. TBE is largely absent in central Poland, and the TBE spike in NE Poland was no greater in the regions closest to Lithuania than in other parts of this large country (Figure 2). Plausible mechanisms for spatial autocorrelation in this disease system that could explain the sharp increase in incidence in 2009 (as distinct from simply the presence of TBE) are human habits and conditions associated with the geography of poverty due to history, or geographically coherent weather anomalies.

The hypothesis concerning the effect of the weather was tested and discarded. Across all 14 countries included in this analysis, neither temperature nor precipitation during the tick activity periods in 2009 or 2008 was unusual. Moreover, Latvia, Lithuania and Poland did not differ from the other countries in this respect. This contrasts with 2006 when both temperature and precipitation were significantly different from average (warmer and drier), particularly in those countries (Switzerland, Germany, Czech Republic and Slovenia) that showed a spike in TBE incidence in that year (Ref 8 and this study), and in ways that encouraged outdoor activity and thus greater contact with ticks [8]. In 2003, however, weather conditions were very close to the 21-year norm in the Baltic countries and Poland where there were spikes in TBE whose cause has not yet been investigated. The conclusion is that marked variation in annual TBE incidence can be, but is not always, driven by changes in the weather; it was not in 2009.

Rather than with the weather, the increase in TBE in 2009 was correlated with differential socio-economic contexts within which increased unemployment was induced by the recent recession. This context, which changes rather slowly (relative food expenditure, for example, barely changed between 1999 and 2005, the most recent index available), determines susceptibility to risk, while increased unemployment triggered a sudden increase in risk. The greater increase in TBE amongst the unemployed in Lithuania is consistent with this. In other words, only specific pre-existing socio-economic conditions give rise to the potential for certain countries to be at risk of an abrupt increase in TBE incidence during a recession accompanied by unemployment. Under-pinning this statistical result are several plausible mechanisms whereby economically disadvantaged people may be less protected against infection and/or be brought into greater contact with infected ticks in their forest habitat.

Two anti-TBE vaccines ('FSME Immun™' produced by Baxter and 'Encepur™' produced by Novartis) are effective, but their cost may be inhibitive for the poorest people. Vaccination coverage (at least one dose) has increased over recent years, reaching 39% in Latvia by 2008 due to a vigorous campaign started in 1998, but only 9% in Lithuania and 18% in Estonia (unrecorded in Poland), in line with the range in most other European countries. Lack of personal exposure, awareness, knowledge, convenience and money are the most common reasons for not getting vaccinated, and one in three do not complete the 3-dose course in Latvia and Lithuania (GfK Group survey, reported by Astrid Essl, 12th annual ISW-TBE meeting, Vienna, January 2010). Low economic status was also identified as a negative factor in TBE vaccination in Latvia in 2000 [3,15]. During 2008 and 2009, the cost of vaccination increased by 40% in Lithuania (followed by a decrease of 12% in 2010), which is likely to have had a negative effect on uptake across the whole population, especially in financially straitened times (Milda Zygutiene, Centre for Communicable Diseases and AIDS, Vilnius, Lithuania, and Aukse Mickiene, Kaunas University, Clinic of Infectious Diseases, Kaunas, Lithuania, personal communication). Concomitantly, sales of TBE vaccine decreased by 23% in 2009 and by a further 30% in 2010.

Another possible factor is an increase in the proportion of viral infections that progress to clinical disease (i.e. recorded TBE cases) as a result of poorer nutrition in impoverished households. This proportion is generally rather low [16], but symptoms are more severe, and therefore recorded incidence disproportionately high, in immunologically compromised patients, as illustrated by the elderly [17,18]. The same might apply to people under stress economically and, as a result, physiologically and/or emotionally.

Any activity that brings people into closer contact with the tick-infested forests for their earned or subsistence livelihood would increase the risk of TBE infection. This could include forestry or simply gathering wood from forests, but the most significant may be the harvest of forest foods that is associated with higher TBE risk to people in Latvia [3,15] and a higher incidence of TBE in the northeastern voivodeships of Poland [11]. This activity has always been associated more with eastern Europe (and Russia) than western Europe [19,20]. In Poland and Russia, the harvest of such foods for generating additional family earnings is associated with low income [11,21]. Increased harvesting and exposure to ticks is not confined exclusively to the unemployed or even the very poorest, but in Poland, 47% of survey respondents in 2004 gave worse financial status of the family as the main reason for increased harvest, while for better off families it represents recreation [11,21]. In Finland, in contrast, mushroom harvest is not a necessity related to the income of the picker, but rather a luxury for the more highly educated, and so less likely to increase in response to unemployment [22,23].

People living in economic uncertainty characterized by high levels of in-work poverty would be expected to react rapidly, probably changing their daily activities when faced with economic downturns. Capitalizing on pre-existing habits and market outlets for NWFP, whether for major exports as from Lithuania and Poland [24] or home markets in Latvia (SER personal observations), would be an obvious route, for which there is some evidence. For instance, in Lithuania, the official bulk purchase of fungi from forests increased from c.1300-2700 tonnes annually during 2004-08 to 5666 tonnes in 2009 http://www.stat.gov.lt. Furthermore, the Private Forest Centre of Estonia reports that 2009 was a good year for mushrooms, which has allowed the unemployed, possibly from the recession, to earn money mushroom picking (translated from Estonian) http://www.eramets.ee/?op=body&id=25&art=498#art498. In contrast, in 'western' Europe, reactions to the relatively modest increases in unemployment are less likely to include more frequent visits to tick-infested forests.

## Conclusion

This is the second of two natural experiments in recent years, both of which reveal the immediacy of the impact of disparate factors on TBE incidence acting through changes in human activities, rather than via the slower responses of the natural enzootic cycles of the virus between ticks and wildlife hosts. First, in 2006, differential responses to unusual weather conditions apparently encouraged outdoor activity for recreation and/or mushroom harvest [9], but left tick populations little changed [8]. Secondly, the current global economic crisis appears to include increased TBE incidence in some European countries as one of its less expected effects. This constitutes an unfortunate opportunity to test conclusions from previous descriptive and analytical studies that identified correlations in time and space between the equally sudden and widespread, but sustained, upsurge in TBE incidence in 1993 and human-induced environmental changes and socio-economic shifts, many arising as an unintended consequence of political independence after the fall of communist powers [1,2]. Food expenditure was identified as a significant correlate of that upsurge too. In one way or another, the multi-factorial drivers of these epidemiological changes affected the living conditions of all four partners, the virus, ticks, wildlife hosts and humans. They differed in force in time and space, however, thereby generating the observed heterogeneous patterns in TBE incidence. The point is not that the variability in TBE incidence is always due to economic forces, but that economic forces are one factor amongst several in causing variability, and in 2009 such factors appear to have been particularly powerful in some countries, specifically Latvia, Lithuania and Poland. Vector-borne diseases, and especially tick-borne diseases for which the risk of exposure to infectious agents depends so much on human behaviour [25,26], are prime examples of complex systems; the output of interest, the incidence of human infections, can vary abruptly due to changes in any one of a diverse range of factors, some of them unexpected.

Attempts to limit the risk of TBE infections will be most effective if targeted at high-risk groups, which, on the basis of these findings, appear to include economically disadvantaged people. Furthermore, if this greater risk arises from habitual activities determined by their economic status, such people are also least likely to be able to change their behaviour to control that risk. Effective practical steps for public health authorities include the promotion of awareness and protection against tick bites, and making either of the two anti-TBE vaccines both accessible and affordable. Given the moderate to severe neurological sequelae that adversely affect the ability to work in c.30-50% of TBE patients [27], subsidization would surely outweigh the costs of such disablement.

## Competing interests

SER attends the annual meetings of the International Scientific Working Group on TBE, fully funded by Baxter.

## Authors' contributions

The study arose out of previous work by SER, who wrote the manuscript. ERG carried out the data analysis, and contributed significant ideas, interpretation and improvements to the final manuscript.
